# Peri-implantitis, Risk of Recurrence and Implant Loss in Soldiers with Stage III/IV Periodontitis during 20–30 Years of Supportive Periodontal Therapy (SPT)

**DOI:** 10.3290/j.ohpd.b5884987

**Published:** 2024-12-18

**Authors:** Felix Wörner, Thomas Eger, Adrian Kasaj, Benjamin Ehmke

**Affiliations:** a Felix Wörner Periodontist and Assistant Medical Director, Department of Dentistry-Periodontology, Bundeswehr Central Hospital Koblenz, Koblenz, Germany. Methodology, investigation, software, data curation, wrote original draft.; b Thomas Eger Periodontist and former Clinical Director of Dentistry-Periodontology, Bundeswehr Central Hospital Koblenz, Koblenz, Germany. Conceptualisation, methodology, investigation, wrote and reviewd the manuscript, project administration, funding acquisition.; c Adrian Kasaj Periodontist and Deputy Head of Clinic of Periodontology and Conservative Dentistry, University Hospital Mainz, Mainz, Germany. DDS, DMD, PhD: Conceptualisation, hypotheses, edited and proofread the manuscript.; d Benjamin Ehmke Periodontist and Clinical Director, Clinic for Periodontology and Conservative Dentistry, University Hospital Münster, Münster, Germany. Conceptualisation, hypotheses, edited and proofread the manuscript.

**Keywords:** dental implants, peri-implantitis, periodontal treatment, periodontitis

## Abstract

**Purpose:**

To help fill the knowledge gaps regarding the long-term effectiveness of peri-implantitis therapy, this retrospective study of soldiers with treated severe periodontitis (stage III gen. / IV) who had been undergoing adherent SPT for at least 20 years aimed to determine the frequency of peri-implantitis and patient-related risk factors for this, as well as the 10-year survival rates of dental implants under peri-implantitis therapy.

**Materials and Methods:**

The observation period was between 1993 and 2023. All patients were referred by their military dentists for specialist dental training and underwent systematic periodontal therapy. A multi-stage peri-implantitis treatment concept was used.

**Results:**

In 86 (31%) of 276 patients (total treatment time 23.6 ± 2.8 years, 53.1 ± 20.4 SPT sessions), 296 implant restorations were performed to close gaps or lengthen rows of teeth. In 29 (33%) of the implant patients, peri-implantitis developed on 25% of the implants. As a result, 11% of all implants were lost within 10 years due to peri-implantitis. Peri-implantitis led to implant loss in 59% of affected patients and 45% of implants. The survival time of implants lost later was 8.4 ± 6.2 years. Peri-implantitis and implant loss rates increased statistically significantly in stage IV periodontitis as well as in heavy smokers. Patients with implant loss and peri-implantitis had received systemic antibiotics due to periodontitis recurrence statistically significantly more frequently than patients without peri-implantitis and without implants during the ≥ 20-year SPT (p < 0.05).

**Conclusions:**

Based on the present results, the early extraction of teeth during SPT in patients with treated generalised periodontitis stage III and stage IV plus replacement with implants is not advantageous.

Peri-implantitis has been defined as a “peri-implant biofilm-associated pathological condition, occurring in tissues around dental implants, and characterised by inflammation in the peri-implant mucosa and subsequent progressive loss of supporting bone”.^2^ Clinically, peri-implantitis sites exhibit inflammation, bleeding on probing (BOP) and/or suppuration (SUP), increased probing depths (PD) and/or recession of the mucosal margin, in addition to radiographic bone loss.^2^

Determining peri-implant health requires a clinical examination to confirm the absence of peri-implant soft-tissue inflammation.^42^ The primary etiological factor for peri-implantitis onset and progression is the accumulation of a peri-implant plaque biofilm. From a biological perspective, the goal is to shift from an anaerobic environment associated with inflammation to an aerobic ecosystem found under healthy conditions.^38^ A good level of evidence exists for the most likely factors in implant therapy that may lead to peri-implantitis (i.e., inappropriate patient selection, insufficient periodontal therapy, lack of diagnosis and management of peri-implant mucositis, erratic supportive peri-implant/periodontal therapy). In contrast, for others (i.e., wrong implant placement, poor postoperative care, inadequate prosthetic reconstruction, lack of assessment and management of peri-implant soft-tissue deficiencies), there is little scientific evidence.^37^ Experimental peri-implant mucositis was established in humans after an undisturbed plaque accumulation phase of 3 weeks.^41^ After an observation period of 5 years, the conversion of clinically manifest peri-implant mucositis to peri-implantitis without therapy was 43.9%. Regular preventive treatment reduced the incidence in the control group to 18.0%.8 If left untreated, the progression of peri-implantitis leads to implant loss.^46^

The prevalence of peri-implantitis was determined in meta-analyses of 47 studies with 19.8% of implants (CI: 14–30%) and 43% (CI: 32–54%) at the subject level.^11,23^ Longer examination periods of the studies (average 2.04 years) and higher implant-to-subject ratio (average 4.76) were associated with subject-based peri-implantitis prevalence.^23^ An implant disease risk assessment evaluates the following parameters: history of periodontitis, percentage of implant and tooth sites with positive bleeding on probing, number of sites with pocket depth ≥ 5 mm at implants and teeth, periodontal bone loss in relation to patient’s age, patient’s susceptibility to periodontitis, compliance with supportive peri-implant care (SPIC) interval ≤ 5 months up to 6 months, restorative margin of the implant prosthesis to the marginal bone crest and the assessment of cleanability.^16^ This is for patients with previous generalised periodontitis stage III or IV, with residual periodontal pockets ≥ 5 mm and an unfavourable relationship between bone resorption and age in the highest risk group. Other risk factors for peri-implantitis, such as tobacco consumption or the width of the attached mucosa around the implants, are not taken into account.^40^

For the management of peri-implantitis, a step-by-step approach may be appropriate, similar to what has been suggested for the treatment of periodontitis.^7,9,24,44^ Non-surgical treatment methods lead to a statistically significant improvement in the clinical parameters investigated after 6 to 12 months. The treated sites often show residual probing depths and bleeding upon probing.^43^ A possible minor favourable effect of adjuvant systemic antibiotic therapy was pointed out in a meta-analysis.^32^ The purpose of a surgical approach in managing peri-implantitis is to provide access to the implant to facilitate surface decontamination.^12^ Studies demonstrate that the progression of peri-implantitis occurs in the presence of clinical signs of inflammation and is manifested through the reduction of peri-implant bone levels.^4,5,21^

Hence, the achievement of pocket closure (≤5 mm) has been regarded as the primary therapeutic endpoint to prevent disease progression.^19,48,50^ Furthermore, SPIC is pivotal in maintaining hard and soft tissue stability over the long term.^52^ Several clinical studies included in a systematic review with follow-up periods of 1-10 years found the rate of compliers with SPIC to be variable (3.3%–86.8%), with smoking habit and a history of periodontal disease being critical predictors of the level of compliance.^1,16^

There is a lack of knowledge on the long-term effectiveness of peri-implantitis therapy. However, explantation should always be performed in the event of implant loosening, irreversible technical complications, complex implant designs (e.g., hollow cylinders), resistance to therapy, or spread of the infection to neighbouring anatomical structures.^18^

Peri-implant diseases, especially peri-implantitis, represent a growing public health problem due to their high prevalence and the associated consequences (implant and implant-supported prosthesis loss), including dental care costs, which are substantial.^18^

## Purpose

The purpose of this retrospective study of soldiers with treated severe periodontitis (stage III gen. / IV) who had been undergoing adherent SPT for at least 20 years was to determine the incidence of peri-implantitis, patient-related risk factors (diabetes mellitus, tobacco consumption, patient compliance, adjunctive antibiotics for periodontal therapy, tooth loss), the duration of retention of implants with peri-implantitis and the 10-year survival rates under peri-implantitis treatment.

## MATERIALS AND METHODS

### Patients

The observation period was from 1993 to 2023. All patients were referred by their military dentists for specialist dental treatment and underwent systematic periodontal therapy.

The present study was based on a questionnaire to record smoking behaviour at baseline and the end of the study, clinical periodontal examinations, and patient-dentist consultations. To ensure that no additional diseases existed that would have an unfavourable effect on the treatment of periodontitis, the patients were asked to undergo a blood test by their military doctor at their home base to rule out cardiovascular diseases, diabetes, HIV infection, leukaemia and the determination of inflammatory parameters as part of a blood count.

Patients were categorised based on their self-reported smoking history. Non-smokers were those who had never smoked or had quit at least 5 years before treatment. All other patients were classified as current or former smokers. Among the smokers, further differentiation was made based on their daily cigarette consumption, with those smoking up to 10 cigarettes per day (S1) and those smoking more than 10 cigarettes per day (S2) forming distinct groups.

Patients were categorised according to their socioeconomic status using educational level as a proxy: (i) up to middle school plus professional training, (ii) middle school plus professional training and specialised independent military rank, (iii) high school and (iv) university education.

Compliance measures how well patients adhere to medical recommendations.^47^ Patients attending 80% of all SPT visits within the recommended time of 1 month were designated as fully adherent.^10^ Patients attending 50–80% of all SPT visits and extending the recommended intervals by 1 month but not more than 100% were considered partially adherent. If patients had extended the recommended SPT interval at least once by over 100%, they were considered non-adherent (e.g., the recommended SPT interval was 6 months, and the patient returned for SPT after 13 months)13 and were excluded from the study.

Retrospectively, each patient was assigned a baseline diagnosis of generalised periodontitis stage III or IV according to the current classification of periodontal diseases.^31^

All patients underwent systematic periodontal therapy with motivation for effective oral hygiene, non-surgical periodontal therapy as “full-mouth deep scaling and root planing” (FMDSRP) using air scalers and hand instruments in one session under infiltration anaesthesia, re-evaluation of the periodontal examination parameters, and follow-up cleaning after 6 weeks. In the case of an indication for adjunctive systemic antibiotic treatment during periodontal treatment, this was carried out with amoxicillin (3x500 mg/day) + metronidazole (3x400 mg/day) for 1 week.^44^ Adjunctive to the subsequent oral hygiene measures, the patients applied 1% chlorhexidine gel (Chlorhexamed Mundgel, GSK; Munich, Germany) at home twice daily for 2 weeks. Further treatment sessions were conducted every 6 weeks as part of the SPT until no bleeding probing depths were ≥ 6 mm. The observation period started with baseline (BL), defined as the start of SPT. All patients’ periodontal findings were re-evaluated 6–12 weeks after non-surgical or surgical periodontal therapy. If PPD ≥ 6 mm with positive bleeding or pus leakage after probing was present, systemic antibiotics were used, and, if necessary, periodontal surgery was performed. SPT intervals were assigned according to individual patient criteria (every 3, 4, 6 or 12 months).^22^ Patients with periodontitis stage III/IV at BL but without implant restorations during the observation period comprised the control group.

### Implant Therapy and Restorations

In all patients, implant treatment was performed after periodontal therapy during SPT. All implants were placed at the soft-tissue level and were cleanable (1993–2003: Nobel Biocare Branemark System MK II / MKIII TiUnite; Götenborg, Sweden; 2002–2023: CAMLOG Screw-Line Promote: Wimsheim, Germany).

Baseline probing on implants started within 3 months of prosthesis insertion. A probe with a 0.5-mm diameter tip and a light probing force (0.2 N) was used; circumferential peri-implant probing depths (ideally at 6 sites), and BOP were recorded. In addition, the authors recommended an intra-oral radiograph after physiological remodelling to document marginal bone levels.

Peri-implant health status was assessed at each clinical examination with peri-implant probing to determine the presence of BOP and monitor changes in PD and mucosal margin level. SPIC was recommended in patients who had healthy peri-implant tissues to reduce the risk of incident peri-implant diseases, emphasising to the patient the importance of their adherence to SPIC visits and home care.^6^

If PD increased in conjunction with BOP/SUP at subsequent visits, an intra-oral radiograph was recommended to evaluate the marginal bone levels.^2,20,25,34,45^

Peri-implantitis was diagnosed according to the contemporary definition at the time of diagnosis, in this case, the early 1990s.^26,27^ The diagnosis was verified in accordance with the European Federation of Periodontology classification at that time. In the absence of initial findings at the time of insertion of the prosthetic superstructure, peri-implantitis was defined in the presence of clinical signs of inflammation, bleeding after probing, suppuration if present, a probing depth of 6 mm and a radiological bone level ≥ 3 mm apical to the imaginary rough-smooth border on the implant.^2,34^

Multi-stage peri-implantitis treatment was carried out as part of the SPIC. The main objective of the non-surgical step of peri-implantitis treatment is to control peri-implant biofilms and inflammation. Therefore, the central intervention was supra- and sub-marginal mechanical instrumentation. For dental implant biofilm removal, stainless steel area-specific curettes, ultrasonic/sonic instruments, rubber cups, and air-polishing devices with glycine powder were used alone or in combination.

The patient was recommended to brush dental implants and teeth twice daily using either manual or re-chargeable power brushes and once daily using interproximal brushes of appropriate size. The patient demonstrated this to the oral healthcare professional and was periodically reinforced.

At the implant level, residual probing depths ≤ 5 mm with no BOP at more than one point and no suppuration were used as therapy endpoints. We recommended considering additional treatment if the endpoints were not achieved 3 months after the non-surgical treatment phase.

Systemic antibiotics were administered for the management of peri-implantitis, limited to cases with deep pockets ≥8 mm and extensive suppuration: metronidazole 500 mg/8 h/ 7 days^3^ or azithromycin 500 mg on the day of surgery and 250 mg, once per day, for 4 additional days^15^ if the patient did not support metronidazole therapy.

Target sites for surgical treatment were those presenting with persistent signs of pathology after non-surgical therapy that were deep pockets together with BOP/SOP.^12,21^ In addition to flap elevation and removal of inflamed tissue, a standard surgical procedure included cleaning/decontaminating the implant surface, for example using small pieces of gauze soaked in saline and removing mineralised deposits with curettes. Additional procedures in the surgical treatment of peri-implantitis may have included the management of peri-implant osseous defects using reconstructive approaches.^35^

In patients treated for peri-implantitis, SPIC at 3–4 months intervals for the first 12 months was recommended to prevent the recurrence of peri-implantitis and implant loss.^36,52^ Frequency was thereafter tailored according to patient-, implant- and restoration-based risk factors.^52^ Implants that developed disease recurrence required additional surgical procedures or could lead to implant loss.^4,12^

### Statistical Analysis

The primary outcome variables of this study – peri-implantitis and implant tooth loss after active periodontal and implant treatment (APT) – were assessed by comparing the first and last (20-30 years later) SPT examinations. The patient served as the statistical unit.

The data were analysed using descriptive statistics. The determination of patient- and implant-specific risk factors was analysed using univariate ANOVA (SPSS 23, IBM; Armonk, NY, USA). The three groups (peri-implantitis, implant loss, and the control group) were not matched for specific characteristics.

Frequencies of gender, smoking status, socioeconomic status, and diabetes mellitus were calculated and compared using a chi-squared test between the control and implant treatment groups. Standard mean deviations of age, number of teeth at baseline, tooth loss in general, and implant loss were calculated and compared using ANOVA between the control and implant treatment groups.

Using multiple linear regression, factors that influenced the dependent variables of peri-implantitis and implant loss in relation to the number of teeth present at first SPT were identified. The following independent variables were entered into the models: treatment group (tooth loss: control), gender, age, socioeconomic status, diagnosis at the initiation of therapy (stage III generalised, stage IV localised, stage IV generalised), diabetes mellitus, adjunctive antibiotic treatment, and nicotine consumption (current smoker vs non-smoker and former smoker). Third molars were excluded from the analysis.

### Ethics Statement

Clinical questionnaires, examinations, and multiple patient-dentist discussions were used to conduct the present investigation. The retrospective study was conducted at the Bundeswehr Medical Service Academy. In full accordance with ethical principles, the guidelines of the Helsinki Declaration were followed, and the Regional Ethics Review of the State Chamber of Physicians of Rhineland-Palatinate in Germany (837.486.13/9171-F) approved the study (March 28, 2014). Subjects were informed that they could leave the study at any time without consequence. All participants were military personnel at baseline. Written informed consent was obtained from all subjects involved in the study after providing referred patients with written informational material.

## RESULTS 

After the patients received information and explanations, they gave written informed consent. 276 voluntary periodontitis patients were included, with a proportion of women of 21.7% (n: 60). The proportion of women among patients with stage IV periodontitis was 30% and for stage III periodontitis 20.2%.

**Fig 1:**
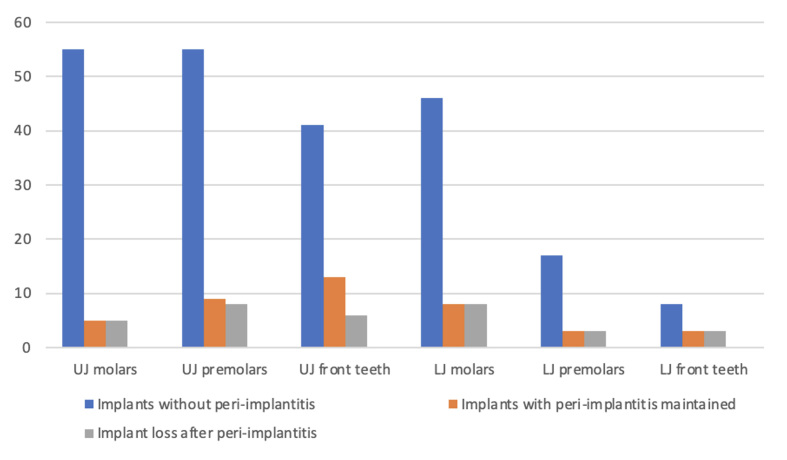
Total number of prosthetically restored implants, including implants retained after peri-implantitis therapy and implant losses by implantation region.

**Fig 2:**
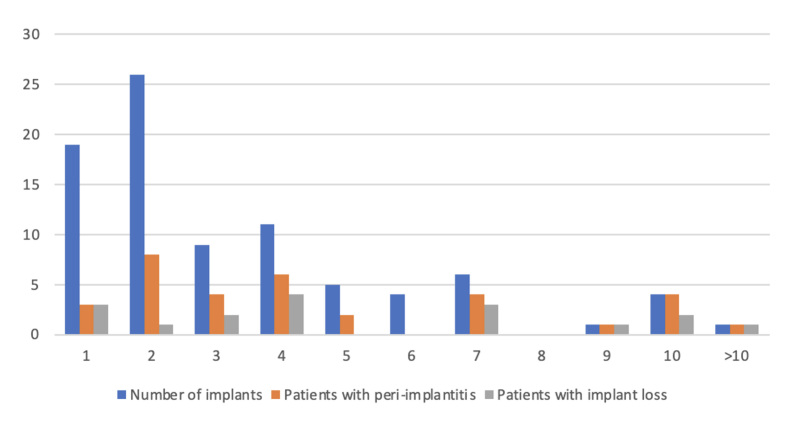
Number of implants inserted per patient and proportion of patients with peri-implantitis and implant loss.

In 86 (31%) patients with treated generalised severe periodontitis stage III or stage IV in adult 20- to 30-year SPT (total treatment time 23.6 ± 2.8 years, 53.1 ± 20.4 SPT sessions), 296 implant restorations were performed to close gaps or lengthen rows of teeth. At BL, there were no differences in age, gender distribution or education between the patients who received implants and those who did not (Table 1). There were 197 (66.5%) implantations in the maxilla and 99 implantations in the mandible (Fig 1). 3.1 ± 2.4 implants were placed per implant patient (Fig 2). At the beginning of the study, 8 patients had 22 implants, of which 7 implants were lost over the course of the SPT following peri-implantitis. Among the implant patients, there were statistically significantly more smokers, patients with generalised stage IV periodontitis, and they were less likely to be diabetic. Implant patients had completed statistically significantly fewer SPT sessions (Table 1).

**Table 1:** Anamnestic data of patients with implant restorations compared to the control group without implant restorations at baseline

	Implant patient (%)	No implant patient (%)	p
Number of patients	86	190	
Men n	64 (74)	152 (80)	
Age at BL (years)	44.1 ± 9.6	44.0 ± 9.4	
Smoker BL n	49 (57)	83 (44)	< 0.05
Diabetes n	5 (6)	27 (14)	< 0.05
School education at BL n General secondary school	2 (2)	2 (1)	
Intermediate secondary school	16 (18)	53 (28)	
High school	32 (37)	58 (31)	
University	36 (42)	77 (41)	
Periodontitis gen. stage III n	54 (63)	154 (81)	< 0.005
Periodontitis loc. stage IV n	14 (16)	16 (8)	
Periodontitis gen. stage IV n	18 (21)	20 (11)	< 0.005
Years of treatment	23.7 ± 2.7	23.6 ± 2.9	
SPT treatment sessions	53.1 ± 20.4	57.8 ± 17.0	< 0,05
Number of teeth BL	23.5 ± 4.1	24.8 ± 3.7	< 0.05
Lost teeth (mean)	5.6 ± 4.8	2.4 ± 3.8	< 0.05
Patients with tooth loss	84 (98)	117 (62)	< 0.000
Number of implants BL (n = 8)	2.8 ± 1.6		
Number of implants end	3.1 ± 2.4		
Patients with peri-implantitis	29 (33)		
Implants per patient with peri-implantitis	2.4 ± 2.3		
Patients with implant loss	17 (20)		


Patients with peri-implantitis were statistically significantly more likely to be smokers and to have stage IV periodontitis than the other implant patients. They also lost statistically significantly more teeth during the 20- to 30-year SPT, received more antibiotics due to periodontitis recurrences, and received more implant restorations than patients without peri-implantitis. There was no difference between the two groups regarding the proportion of patients with complete and partial compliance with the perceived SPT sessions (Table 2).

**Table 2:** Treatment results for implant patient groups with and without peri-implantitis

	Peri-implantitis patient (%)	Implant patient (%)	p
Number of patients	29	57	
Number of teeth BL	21.9 ± 5.5	24.4 ± 2.9	< 0.05
Lost teeth (mean)	7.2 ± 5.4	4.7 ± 4.3	< 0.05
Patients with tooth loss	27 (93)	57 (100)	
Diabetes	1 (3)	4 (7)	
Implants with peri-implantitis n	41		
Full adherent	17 (59)	32 (56)	
Semi-adherent	12 (41)	25 (44)	
Smoker BL n (%)	21 (72)	28 (49)	< 0.05
Of which smokers > 10 cigarettes/day	17 (59)	10 (18)	< 0.001
Smokers End n (%)	15 (52)	16 (28)	< 0.05
Of which smokers > 10 cigarettes/day	9 (31)	4 (7)	< 0.05
Periodontitis gen. stage III n (%)	14 (48)	40 (70)	< 0.000
Loc. stage IV n (%)	2 (7)	12 (21)	
Gen. stage IV n (%)	13 (45)	5 (9)	< 0.000
Years of treatment	23.7 ± 3.1	23.1 ± 2.9	
SPT treatment sessions	51.8 ± 21.1	53.8 ± 20.1	
Number of implants end	3.9 ± 3.1	2.8 ± 2.0	< 0.05
Systemic antibiotics	2.64 ± 1.50	1.63 ± 1.91	< 0.05
Patients with systemic antibiotics use	28 (96)	54 (75)	< 0.05


Implant-loss patients were statistically significantly more likely to be heavy smokers (S2) and had higher tooth loss at BL than patients with peri-implantitis without implant loss. They were also considerably more likely to receive systemic antibiotics due to periodontitis recurrence (Table 3).

**Table 3:** Treatment results in patients with peri-implantitis and patients with implant loss.

	Patients with implant loss (%)	Implant patients (%)	p
Number of patients	17	69	
Number of teeth BL	20.8 ± 6.3	24.2 ± 3.1	<0.05
Number of implants end	3.9 ± 3.1	2.8 ± 2.0	
Stage IV generalised	8 (47)	10 (14)	<0.05
Survival years of lost implants	8.4 ± 6.2		
Systemic antibiotics	2.9 ± 1.6	1.8 ± 1.8	<0.05
Tobacco consumption BL Never smokers	2 (12)	20 (29)	
Former smokers	3 (18)	12 (17)	
Smokers < 10 cigarettes/day	3 (18)	19 (28)	
Smokers > 10 cigarettes/day	9 (53)	18 (26)	<0.001
End of tobacco consumption Never smokers	2 (12)	20 (29)	
Former smokers	5 (29)	28 (41)	
Smokers < 10 cigarettes/day	3 (18)	15 (22)	
Smokers > 10 cigarettes/day	7 (41)	6 (9)	< 0.05


In 29 (34%) of the soldiers with implant restorations, peri-implantitis developed on 74 (25%) of the implants. Non-surgical peri-implantitis therapy was performed on all affected implants. Surgical peri-implantitis therapy was performed on 50 implants 3-6 months later. As a result, 33 (11%) of the 296 implants were lost within 10 years due to peri-implantitis during SPT/SPIC (Fig 1). Peri-implantitis led to implant loss in 59% of the affected soldiers and 45% of the implants (Fig 2). The survival time of implants lost later was 8.4 ± 6.2 years.

Thirteen (72%) of the 18 patients with treated stage IV periodontitis and implant restorations developed peri-implantitis on 48% of their 99 placed implants and lost 46% of them.

Nine (69%) of the 13 patients with tobacco consumption > 10 cigarettes at BL (S2) developed peri-implantitis on 41% of their implants and subsequently lost 55% of them. Among the 18 patients with tobacco consumption ≤ 10 cigarettes/day (S1), 6 (33%) of the soldiers developed peri-implantitis on 23% of their implants and lost 50% of them.

## DISCUSSION

In two meta-analyses, the average implant survival rate at a follow-up >5 years for periodontitis patients of varying severity was 91.7% at the implant level and 94.7–86.4% at the patient level.^6,11^ A statistically significant negative relationship was shown between the prevalence of peri-implantitis and the threshold for marginal bone loss.^11^

In the adult patients with treated, severe periodontitis who were in 20–30-year SPT and examined in our study, the implant survival rate over 10 years was 89% at the implant level and 80% at the patient level. This lower survival rate may be due to the long treatment period, the treatment protocol described, the clearly defined high severity of the previously treated periodontitis with a higher risk of tooth loss, as well as various systemic and periodontal risk factors.

Important risk factors/indicators have been identified, including a history of severe periodontitis, poor plaque control and no regular supportive peri-implant care (SPIC) following implant therapy. A recent 20-year follow-up study on 62 periodontally compromised patients’ non-compliance with SPC was the most important, statistically significantly greater risk for peri-implantitis (odds ratio 14.3; confidence interval 1.8–32.9).^36,39^ Less conclusive evidence was found for diabetes^30^ and smoking or local factors and maintenance.^18,46,47^ A long-term retrospective study indicated that the stages and grades of periodontitis are risk indicators for peri-implant diseases. Peri-implant disease was more common in patients with stage IV periodontitis.^54^

Our results suggest a correlation between smoking > 10 cigarettes per day, diagnosis of generalised periodontitis stage IV (number of teeth after AIT) and the development of peri-implantitis.

Since the 1980s, the proportion of smokers in the adult population in Germany has been falling slightly. In 2001, 44.5% of young adults aged 18 to 25 smoked. In 2021, 29.85% still did (Federal Ministry of Health, Berlin www.bundesgesundheitsministerium.de/service/begriffe-von-a-z/r/rauchen#c29700, accessed: 10 May 2024). Per capita consumption of cigarettes has been declining in recent years. In 2023, consumption fell to 764 cigarettes per inhabitant (2022: 785; Federal Statistical Office [ed.] [2024]): Sales of tobacco tax statistics. Wiesbaden. www-genesis.destatis.de/genesis/online31&code=73411#abreadcrumb, accessed: 06/03/2024).

The proportion of smokers at baseline in our study was 48% and 57% among patients who received implants. 75% of patients with peri-implantitis were smokers. Tobacco use was a statistically significant risk factor for the development of peri-implantitis and implant loss. However, smokers were more likely to receive implants than non-smokers.

The prevalence of type 2 diabetes mellitus in Germany is currently 7.4%.^33^ 12% of the patients in our study developed diabetes in the SPT period of 20–30 years. As part of the SPT, the current HbA1c value was asked of each diabetic patient, and the importance of good glycaemic control for general health, periodontal stability, and prevention of tooth loss was confirmed. The risk factor diabetes for peri-implantitis in adult patients whose periodontitis is treated long-term can, therefore, not be confirmed by our results.

Patients with implant loss and peri-implantitis were statistically significantly more likely to have periodontitis recurrences during the SPT period when adjunctive antibiotics were used in addition to non-surgical periodontitis treatment. The benefit of systemic antibiotics in peri-implantitis treatment as an adjunct to sub-marginal instrumentation alone was statistically significant and clinically relevant. It tended to be more pronounced in cases with initially deeper lesions and to improve over time up to 1 year.^3,14,51^ The benefit size may allow achievement of the stipulated treatment endpoints in many cases, thus obviating surgical intervention.^32^ Therefore, the clinical recommendation that antibiotics cannot be recommended as a routine is based on the general principles of antibiotic stewardship and the public health objective of limiting unnecessary use of antibiotics in dentistry.

The implant patients in our study group completed statistically significantly fewer SPT sessions than patients without implants. 57% of patients with implant restorations were fully adherent to SPT over the > 20-year period. There were no differences in compliance and SPT sessions between the implant patient groups with and without peri-implantitis. The peri-implantitis patients completed 1.8 ± 0.5 SPIT/year (52% stage IV periodontitis) with 296 implants. The patients with implant loss had more frequent periodontitis recurrences and received adjunctive systemic antibiotics (2.9 ± 1.6) with additional non-surgical anti-infective and periodontal surgical therapy sessions. All periodontal findings were re-evaluated 6–12 weeks after non-surgical or surgical periodontal therapy in all patients and had additional active periodontal therapy sessions. This confirms the study results of Monje et al28 on compliance and SPT/SPIC sessions in their peri-implantitis study population, with a peri-implantitis rate of 43.8% during a follow-up of 35.3 ± 16.3 months after peri-implantitis treatment. Comprehensive information provided before peri-implantitis treatment regarding the importance of adhering to SPIC after peri-implantitis treatment to achieve/maintain peri-implant health resulted in 57.2% regular compliance rate ≥ 2 SPIC/year in a group of 161 patients (43.7% stage IV periodontitis) with 1079 implants.^28^

Surgical peri-implantitis therapy was performed on 50 of the 74 implants with peri-implantitis due to recurrence/progression of peri-implantitis 3–6 months after non-surgical treatment. The survival time of subsequently lost implants was 8.4 ± 6.2 years. Peri-implantitis led to implant loss in 59% of the affected soldiers and 45% of the implants (Fig 2). This agrees with other studies. Over 5-year observation periods, disease recurrence/progression of peri-implantitis was observed at 32%–44% of treated implants. Corresponding implant loss was low in the short term but, after 5 years, it ranged from 14% to 21%.^12,21^ In our study, 16 of the 17 patients (94%) with implant loss and 32 of the 33 patients with peri-implantitis had at least one periodontitis recurrence in the 20-year period. In the two evaluations covering longer follow-ups ≥ 5 years, loss to follow-up exceeded 20%.^4,16,17^ The higher recurrence/progression rate of peri-implantitis of the patients in our study may be due to the implant follow-up of > 10 years, the selection of patients with stage III gen. / stage IV periodontitis, and the exclusion of non-compliant patients.

Patients who developed peri-implantitis had previously received statistically significantly more implants in our study than patients from the group without peri-implantitis (3.9 ± 3.1 vs 2.8 ± 2.0) (Fig 2). This confirms the results of the meta-analysis by Lee et al.^23^ These had longer study periods (average of 2.04 years), and higher implant-to-subject ratios (average of 4.76 implants) were associated with subject-based peri-implantitis prevalence.^23^

The tooth loss rate of patients with implant-supported restorations was 0.19 teeth/year, and that of patients with peri-implantitis was statistically significantly higher at 0.30 teeth/year during the study period. Patients with implant restorations had substantially higher tooth loss during their > 20-year SPT compared to patients without implant restorations (5.6 ± 4.8 vs 2.4 ± 3.8) (Table 1). This could have been determined not only by the previously mentioned risk factors but also by patient preference or prosthetic restorations in cases of pre-existing tooth loss due to the severity of periodontitis at BL. Furthermore, evidence exists that prosthetic factors may also increase the risk of onset/progression of peri-implant diseases.^46^

### Limitations 

At baseline, the patient group in this study consisted of Bundeswehr (Germany army) soldiers of different rank groups and, thus, a lower proportion of women. Soldiers undergo mandatory dental examinations by the military to determine their dental fitness. Dental treatment, on the other hand, is compulsory only for deployment. Implant therapy, including prosthetic treatment, is not associated with additional costs for the soldiers in our study. Thus, when deciding whether to maintain a periodontally compromised tooth or replace it with a dental implant, in terms of cost-effectiveness, implant maintenance cost and the cost associated with treating implant complications should be considered in the civilian health-insurance system.^29^ This cost seems to surpass the cost of treatment and maintenance of periodontally compromised teeth in civilian patients at high risk for peri-implantitis.^49^ Despite the presented risk-dependent implant loss rates of 46–50%, soldiers in SPT with implant-supported restorations for treated stage III and IV periodontitis are fit for deployment with peri-implantitis therapy. The transferability of the results of this long-term (over more than 20 years of SPT) specialist dental study to general dental periodontological therapy requires further clinical studies.

## CONCLUSIONS

Peri-implantitis, implant- and tooth-loss rates increase statistically significantly in stage IV periodontitis as well as in heavy smokers. SPIC programs should include interventions for primary prevention of peri-implant diseases, such as professional supra- and sub-marginal plaque biofilm removal, oral hygiene motivation and coaching, as well as early detection of pathological conditions.

The success of treatment after non-surgical peri-implantitis treatment should be re-evaluated at the latest after 3–6 months. If the pre-established criteria of success are not fulfilled and the affected implant is still deemed to be maintainable, it could be treated again, i.e., surgically.

Long-term studies to compare the effectiveness of different peri-implantitis therapy procedures in the context of SPIC require more than 10 years to assess the potential implant retention in adult patients.

The study results do not support early extraction of teeth during SPT in patients with treated generalised periodontitis stage III and stage IV and replacement with implants.

Peri-implantitis is an irreversible condition; therefore, even after successful peri-implantitis therapy, a diagnosis of “stable“ peri-implantitis is assigned at the particular implant.

## ACKNOWLEDGEMENT

Special thanks for the repeated German-English translations and formal help to Anika Noto-Eger, Toronto, Canada.

## Conflicts of Interest

The authors report no conflict of interest. The funders had no role in the study’s design, the collection, analysis, or interpretation of data, the writing of the manuscript, or the decision to publish the results. The opinions expressed in this article are those of the authors. They cannot be construed as reflecting the views of the Bundeswehr Medical Service, the Bundeswehr at large, or the German Ministry of Defence.

Data Availability Statement: The data presented in this study are available on request from the corresponding author.

Funding: The study was funded by the Bundeswehr Medical Service Academy, Munich, Germany (08K1-S-80 1315,02F4-S-80 1922).
